# Acknowledgement to Reviewers of *International Journal of Neonatal Screening* in 2018

**DOI:** 10.3390/ijns5010006

**Published:** 2019-01-09

**Authors:** International Journal of Neonatal Screening Editorial Office

**Affiliations:** MDPI, St. Alban-Anlage 66, 4052 Basel, Switzerland

Rigorous peer-review is the corner-stone of high-quality academic publishing. The editorial team greatly appreciates the reviewers who contributed their knowledge and expertise to the journal’s editorial process over the past 12 months. In 2018, a total of 37 papers were published in the journal, with a median time to first decision of 20 days and a median time to publication of 49 days. The editors would like to express their sincere gratitude to the following reviewers for their cooperation and dedication in 2018:
Aithal, SreedeviMak, Chloe MiuAl-Dirbashi, OsamaMatern, DietrichAndersson, HansMcGill, James J.Angastiniotis, MichaelMcGrath, Andrew P.Bailey, Donald B.McPherson, BradleyBain, Barbara J.Mercer, KristinaBierle, CraigMoser, Ann B.Bonham, JimNagao, MasayoshiBubbico, LucianoNewton, ValerieButcher, EmmaNicholls, Stuart G.Caprari, PatriziaOlivieri, AntonellaChien, Yin-HsiuOrsini, JoeCornel, MartinaPasquali, MarziaCortés Castell, ErnestoPastores, Gregory M.Coutinho, Maria FranciscaPekrun, ArnulfDaniel, Yvonne A.Poulakis, ZeffieDejaco, DanielPowell, Cynthia M.Dotsikas, YannisPrice, PatriciaEckman, James R.Pulignani, SilviaEngel, Paul C.Ralli, MassimoEtchegary, HollyRausch, Christopher M.Ewer, AndrewRiede, Frank-ThomasFeucthbaum, LisaRinaldo, PieroFuente, AdrianSaadallah, AmalGardner-Berry, KirstySequí-Canet, José MiguelGelb, MichaelSirdah, MahmoudGiraldo, PilarSommerburg, OlafGrosse, Scott D.Sontag, MarciHaas, NikolausStamminger, ThomasHammarström, LennartSteyger, PeterHenderson, MatthewStove, Christophe P.Hinton, Cynthia F.Streetly, AllisonHokanson, John SmithTavakoli, Norma P.Hom, Lisa A.Therrell, BradfordHoppel, Charles L.Tortorelli, SilviaHsu, LewisUilenburg, NoëlleInusa, BabaVan Trotsenburg, PaulJansen, MarleenVan Zanten, Gijsbert A.Jerebtsova, MarinaVeraguth, DorotheKay, Denise M.Von Döbeln, UlrikaKennedy, ColinVona, BarbaraKunz, JoachimWebster, DianneLambert, ChrisWilcken, BridgetLeo, Carlo GiacomoWiley, VeronicaLoeber, J. GerardWilson, CallumLorey, FredYoshinaga-Itano, Christine

